# Supplementary material for: HIV testing uptake and HIV positivity among presumptive tuberculosis patients in Mandalay, Myanmar, 2014-2017
Source: PLoS One. 2020 Jun 18;15(6):e0234429. doi: 10.1371/journal.pone.0234429 (PMC7302489; doi:10.1371/journal.pone.0234429)
Supplement: Attachment — Submitted filename: Plos one manuscript Review comments.docx [file pone.0234429.s002.docx]

**Review comments**

**Reviewer:** Joseph Mugisha Okello

**Title for manuscript:** HIV testing uptake and HIV positivity among presumptive tuberculosis patients in Mandalay, Myanmar, 2014-2017

**General comment**

1. This important manuscript covers an important subject on HIV testing uptake and HIV positivity among presumptive tuberculosis patients. As the authors rightly, say, HIV testing uptake is still low in patients with presumptive TB. Therefore this manuscript generates more literature on this important topic
2. The manuscript is well written, and coherent which makes it easy to read. My only concern is why the authors never followed up the 1770 patients that never tested for HIV. In the discussion, the authors talk about the reasons (from qualitative studies) why patients with TB do not test for HIV. However, since the study setting looks to be organised, it would have been better to follow up the 1770 patients (15%) that never tested for HIV to study the reasons why.

**Specific comments**

1. Data for this study was obtained from a TB program. Although the authors got ethical clearance from the National TB and HIV/AIDS programme and the ethics review committee, there is no mention in the manuscript on whether the authors received informed consent from the study participants/patients.
2. It is important that the authors give some details on how patients co-infected with TB and HIV are managed or how and where they are referred.
3. In addition, the authors should give details on how the program follows TB patients referred to the resident townships for continuation of anti-TB treatment.
