# Supplementary material for: HIV testing uptake and HIV positivity among presumptive tuberculosis patients in Mandalay, Myanmar, 2014-2017
Source: PLoS One. 2020 Jun 18;15(6):e0234429. doi: 10.1371/journal.pone.0234429 (PMC7302489; doi:10.1371/journal.pone.0234429)
Supplement: Attachment — Submitted filename: Response to reviewers_Plos one.docx [file pone.0234429.s004.docx]

Dear Editor,

We thank you for the comments of the reviewers and editor. We have been carefully through the review and have revised our paper accordingly in track change mode. We have attempted to address all the comments of the reviewers and give a point-by-point response. We submit a tracked-changes and a clean version of the paper. The line number stated in response are from tracked changes version. We hope this revised paper may now be acceptable for publication in Plos One.

On Behalf of all authors,

Khine Wut Yee Kyaw

Author of correspondence

**Additional Editor Comments (if provided):**

Thank you for the revisions. The comments have satisfactorily addressed. However there are still several language errors in the manuscript. I suggest the final manuscript is copyedited by a native speaker

Author’s response: Thank you. We have carefully revised the manuscript and all language errors have been reviewed and edited by a native speaker.

While revising your submission, please upload your figure files to the Preflight Analysis and Conversion Engine (PACE) digital diagnostic tool, <https://pacev2.apexcovantage.com/>. PACE helps ensure that figures meet PLOS requirements. To use PACE, you must first register as a user. Registration is free. Then, login and navigate to the UPLOAD tab, where you will find detailed instructions on how to use the tool. If you encounter any issues or have any questions when using PACE, please email us at [figures@plos.org](mailto:figures@plos.org). Please note that Supporting Information files do not need this step.

Author’s response: Thank you. We have ensured that figures meet PLOS requirements by using the provided tools.

**Review comments**

**Reviewer:** Joseph Mugisha Okello

**Title for manuscript:** HIV testing uptake and HIV positivity among presumptive tuberculosis patients in Mandalay, Myanmar, 2014-2017

**General comment**

1. This important manuscript covers an important subject on HIV testing uptake and HIV positivity among presumptive tuberculosis patients. As the authors rightly, say, HIV testing uptake is still low in patients with presumptive TB. Therefore this manuscript generates more literature on this important topic

Author’s response: Thank you for this encouraging comment.

2. The manuscript is well written, and coherent which makes it easy to read. My only concern is why the authors never followed up the 1770 patients that never tested for HIV. In the discussion, the authors talk about the reasons (from qualitative studies) why patients with TB do not test for HIV. However, since the study setting looks to be organised, it would have been better to follow up the 1770 patients (15%) that never tested for HIV to study the reasons why.

Author’s response: We agreed that those patients with presumptive TB should be followed up or HIV testing should be offered on their subsequent visits. . However, our current program practice does not require presumptive TB patients to come for follow-up unless they have other health issues or they develop TB symptoms later on. So we have added a recommendation to conduct a further study to explore the reasons for not having HIV testing among presumptive TB patients. We have included this in the manuscript. [line number 346-347]

**Specific comments**

1. Data for this study was obtained from a TB program. Although the authors got ethical clearance from the National TB and HIV/AIDS programme and the ethics review committee, there is no mention in the manuscript on whether the authors received informed consent from the study participants/patients.

Author’s response: Thank you for this comment. As the study involved routinely collected program data, informed patient consent was waived by both ethical committees. We have included this in the manuscript. [line number 209-211]

2. It is important that the authors give some details on how patients co-infected with TB and HIV are managed or how and where they are referred.

Author’s response: Thank you. We have provided this information in the manuscript. [line number 150-156]

3. In addition, the authors should give details on how the program follows TB patients referred to the resident townships for continuation of anti-TB treatment.

Author’s response: Thank you. We have revised this in the manuscript. [line number 154-156]
